# What is the extent of potentially avoidable admissions amongst hospital inpatients with palliative care needs?

**DOI:** 10.1186/1472-684X-12-9

**Published:** 2013-02-18

**Authors:** Merryn Gott, Clare Gardiner, Christine Ingleton, Mark Cobb, Bill Noble, Michael I Bennett, Jane Seymour

**Affiliations:** 1 School of Nursing, Faculty of Medical and Health Sciences, The University of Auckland, Level 2, Building 505, 85 Park Road, Grafton, Auckland, New Zealand; 2 School of Health and Related Research (ScHARR), The University of Sheffield, Regent Court, 30 Regent Street, Sheffield, S1 4DA, UK; 3 School of Nursing & Midwifery, The University of Sheffield, Barber House, 387 Glossop Road, Sheffield, S10 2HQ, UK; 4 Sheffield Teaching Hospitals NHS Foundation Trust, 14 Claremont Crescent, Sheffield, S10 2TA, UK; 5 St Luke’s Hospice, Little Common Lane, Sheffield, S11 9NE, UK; 6 Leeds Institute of Health Sciences, School of Medicine, University of Leeds, Leeds, LS2 9LJ, UK; 7 School of Nursing, Midwifery and Physiotherapy, University of Nottingham, Queen’s Medical Centre, Derby Road, Nottingham, NG7 2HA, UK

**Keywords:** Palliative care needs, End of life, Avoidable admissions, Inappropriate admissions, Survey, Acute hospital

## Abstract

**Background:**

There is clear evidence that the full range of services required to support people dying at home are far from being implemented, either in England or elsewhere. No studies to date have attempted to identify the proportion of hospital admissions that could have been avoided amongst patients with palliative care needs, given existing and current local services. This study aimed to examine the extent of potentially avoidable admissions amongst hospital patients with palliative care needs.

**Methods:**

A cross sectional survey of palliative care needs was undertaken in two acute hospitals in England. Appropriateness of admission was assessed by two Palliative Medicine Consultants using the following data collected from case notes: reasons for admission; diagnosis and co-morbidities; age and living arrangements; time and route of admission; medical and nursing plan on admission; specialist palliative care involvement; and evidence of cognitive impairment.

**Results:**

A total of 1359 inpatients were present in the two hospitals at the time of the census. Of the 654 consenting patients/consultees, complete case note data were collected for 580 patients; the analysis in this paper relates to these 580 patients. Amongst 208 patients meeting diagnostic and prognostic criteria for palliative care need in two acute settings in England, only 6.7% were identified as ‘potentially avoidable’ hospitalisations. These patients had a median age of 84. Half of the patients lived in residential or nursing homes and it was concluded that most could have received care in this setting in place of hospital.

**Conclusion:**

Our findings challenge assumptions that, within the existing configuration of palliative and end of life health and social care services, patients with palliative care needs experience a high level of potentially avoidable hospitalisations.

## Background

In the UK, 56% [1] of people die in acute hospitals and an estimated 90% experience a hospital admission during the last year of life [2,3]. Whilst recent evidence suggests a slow increase in the proportion of deaths at home in England and Wales amongst people age 85 years and over [4], other predictions based on past trends estimate that only one in ten people in the UK will die at home by 2030 and an expansion of inpatient services by one-fifth will be required to meet patient need [5]. This pattern of care will be costly to the health economy as a whole and, more importantly, may result in end of life experiences that are not in line with individual, family and wider societal views of ‘good dying’ [6]. It is therefore unsurprising that the UK, in line with most other, developed countries is paying increased attention to the appropriate use of acute hospitals for patients with palliative care needs [7].

The stated intention of a number of countries to reduce hospital admissions amongst patients with palliative care needs therefore seems in line with a policy commitment to increase home deaths [6], as well as wider policy goals to shift health care provision from acute to community settings [8]. The End of Life Care Programme informing policy and service development in England [6] argues that the significant resources spent on hospital care for people in the last 12 months of life should be reallocated to high quality community based services. However, the research evidence base for such claims remains weak[9] and greater understanding of the use of the acute hospital for patients dying from conditions amenable to palliative care input is urgently needed to inform policy and practice.

A small number of important studies have already been conducted in the UK in this area. Using a retrospective case review methodology, Abel and colleagues [10] reported that 33% of deaths in hospital could have occurred at home (including residential care homes). Admissions were considered inappropriate if they could have been avoided by the full implementation of the End of Life Care Strategy for England [6], including early identification of the likely disease trajectory, Advance Care Planning, out of hours care provision and adequate nursing care to support death at home. A 2008 National Balance of Care Audit [11] involving expert health professional review of 80 case notes of patients who died in one acute hospital in England concluded that, in 40% of cases, patients did not need to be in hospital to have their medical needs met. For these patients, it was concluded that an alternative place of death could have been achieved if enhanced services to support people to die outside hospital were available; there was recognition that ensuring these services were in place would involve significant additional investment, particularly in inpatient and outpatient specialist palliative care services.

However, there is clear evidence that the full range of services required to support death at home are very far from being implemented, either in England or elsewhere [6]. Crucially, we are not aware of any studies that have attempted to identify the proportion of admissions that could have been avoided given existing and current local service provision. In addition, we are not aware of any previous published studies that have examined the issue of potentially avoidable admissions within this patient population which have adopted the standpoint of the admitting clinician, for whom managing clinical risk and ensuring the safety and wellbeing of the patient is of primary concern. Existing data informing current policy making has been retrospectively gathered from clinical notes after a patient has died, preventing a detailed exploration of the wider context of ‘real life’ decision making within an acute clinical situation. This approach also precludes an exploration of hospital admissions amongst patients who have palliative care needs, but do not die during that admission.

With this context in mind, the current paper presents an analysis of the extent of potentially avoidable admissions amongst inpatients with palliative care needs within two acute hospital settings in England.

## Methods

A comprehensive survey of hospital in-patients was undertaken in two hospitals in England which were selected for socio-demographic diversity. The Sheffield Northern General Hospital (SNGH) serves a largely urban, economically disadvantaged and ethnically diverse area. By contrast, the Royal Lancaster Infirmary (RLI) serves a predominantly white Caucasian semi-rural / remote rural population. For example, 98% of the population of Sheffield are classified as living in an urban area as compared to 48% of the population of Cumbria [12] (the county within which Lancaster is situated). Causes of death in both locations are in line with overall trends for England with the top three leading causes being respiratory disease, cardiovascular disease and cancer [12]. In Sheffield, total spend on end of life care per death and hospice services per death is higher than the national average at £2,803 (national average £525) and £2047 (national average £1096) respectively. In Cumbria, the total spend per death on end of life care services and hospice services is below the national average at £498 and £359 respectively [12].

Both surveys were conducted during 2010, over 11 days at SNGH (total inpatients beds at the time of the census = 1144 ) and 5 days at LRI (total inpatient beds = 413). All in-patient wards, except children’s wards and mother and baby units were included. Further details of methods have been reported elsewhere [13]. A team of 30 trained researchers conducted data collection, with each ward visited by a team of two researchers at some point during the survey period. All patients aged 18 years and over resident on the ward at 9 am that day were eligible for inclusion. Non-English speaking patients were excluded because of a lack of translation services. The approach to the inclusion of patients lacking capacity was developed in line with Mental Capacity Act Guidance guidance [14]. Senior clinicians and relatives or close friends (where available) were consulted to identify patients lacking capacity to consent. For those patients, a personal consultee (relative or close friend) was approached and invited to participate on behalf of the patient. For all patients/consultees who consented to participate in the study, the following data were collected:

1. ***From patients’ hospital case notes***: evidence of palliative care need according to Gold Standards Framework (GSF) prognostic indicator criteria [15]: socio-demographic and diagnostic information; details of co-morbidities; reason for admission; evidence of adoption of a palliative care approach using a list of predefined indicators; number of previous hospital admissions in the previous 12 months; medical and nursing plans on admission;and discharge plans. All data were extracted by researchers with a clinical background in medicine or nursing.

2. ***From interviews with medical staff and nursing staff:***A member of nursing staff and a member of medical staff involved in the patient’s care were invited to participate in the survey, with the patient’s consent. Staff were asked to provide further diagnostic and admission information for the patient, as well as to state whether they believed the patient to have palliative care needs according to a standardised definition [16]; whether they would have been surprised if the patient died within 12 months, or during the current admission; whether they thought the current admission could have been avoided; and whether prognosis discussions had taken place or were planned. Staff were also asked about their personal educational needs in palliative care.

### Potentially avoidable admission data

Judgements regarding whether the hospital admission was potentially avoidable or not were made by two Palliative Medicine Consultants (BN and MB) who reviewed data collected from the case notes of participating patients. Consultants reviewed the notes from the hospital they worked at, as in-depth knowledge of local service provision, configuration and extent of health and social care services for this patient group was considered critical to inform decision- making. In the absence of any validated protocols to identify potentially avoidable admissions [17,18], context specific expert judgement was deemed the most appropriate way to classify admissions. Appropriateness of admission was assessed using the following data collected from case notes: reason for admission; diagnosis and co-morbidities; age and living arrangements; time and route of admission; medical and nursing plan on admission; evidence of palliative care involvement; evidence of cognitive impairment. These data were considered in addition to knowledge of local services and a decision was made whether, at the time of admission and from the standpoint of the admitting clinician, the admission was: ‘appropriate’, ‘potentially avoidable’, or ‘insufficient data to make a decision’. An appropriate admission was defined as an admission necessary due to clinical need, taking into account the patient's circumstances and availability of local community services at the time of the admission. For admissions deemed potentially avoidable, alternatives to hospital admission were noted. To ensure consistency in clinical decision-making between the two Consultants, they double reviewed a random sample of 15% of notes. The level of agreement between clinicians was assessed using a Cohen’s kappa statistic. Ethical approval for the study was granted by Nottingham 1 Research Ethics Committee. Research Governance approval was granted by the relevant NHS Trusts.

## Results

### Patient sample

**Figure 1:**
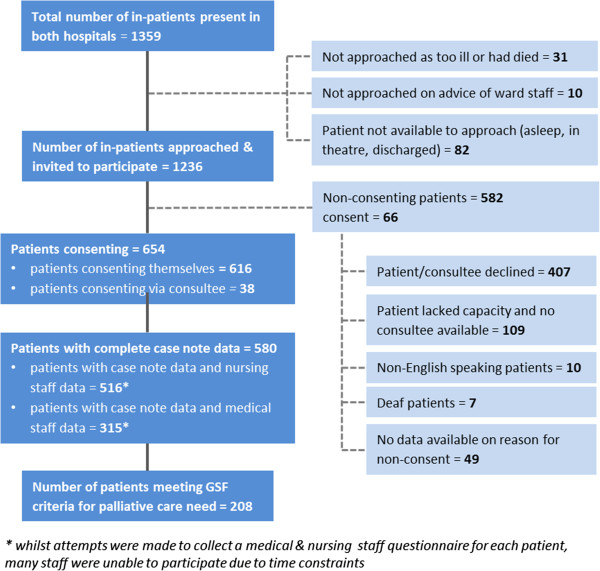
Details of recruitment for hospital in-patients at SNGH and RLI.

A total of 1359 in-patients were present in the two hospitals at the time of the census (1009 patients in Sheffield and 350 patients in Lancaster). Of the 1236 patients who were approached and invited to participate, 654 patients were consented to the study (616 patients consented for themselves, 38 via a consultee). For details of recruitment see Figure 1.

Of the 654 consenting patients/consultees, complete case note data were collected for 580 patients; the analyses in this paper relate only to these 580 patients. There were no significant differences in either age (t = 0.08, p = 0.994) or gender (*χ*^2^ = 0.487, p = 0.488) between these 580 patients and the 74 for whom compete data were not available. In addition to case note data, nursing staff questionnaires were available for 516 patients and medical staff questionnaires were available for 315 patients.

Of the 580 patients in the sample, 208 (35.9%) patients met one or more of the GSF prognostic indicator criteria for palliative care need. Table 1 gives demographic data for the sample of patients with palliative care needs according to GSF criteria. Just over half of these patients were female (53.4%) and the vast majority were older people (85.6% aged ≥60 years), with a median age of 77 years, and an age range of 20 to 103 years.

**Table 1:** Demographic information for patients who have palliative care needs according to GSF criteria (n = 208)


Sex	Male	97 (46.6%)
	Female	111 (53.4%)
Age	Median	77 years
	Age Range	20-103 years
Partnership status	Married	77 (37.0%)
	Single/Divorced	38 (18.2%)
	Widowed	67 (32.2%)
	[No data in notes]	26 (12.5%)
Living arrangements	Lives alone	78 (37.5%)
	Co-habits	97 (46.6%)
	Nursing home or residential care	19 (9.1%)
	[No data in notes]	14 (6.7%)

**Figure 2:**
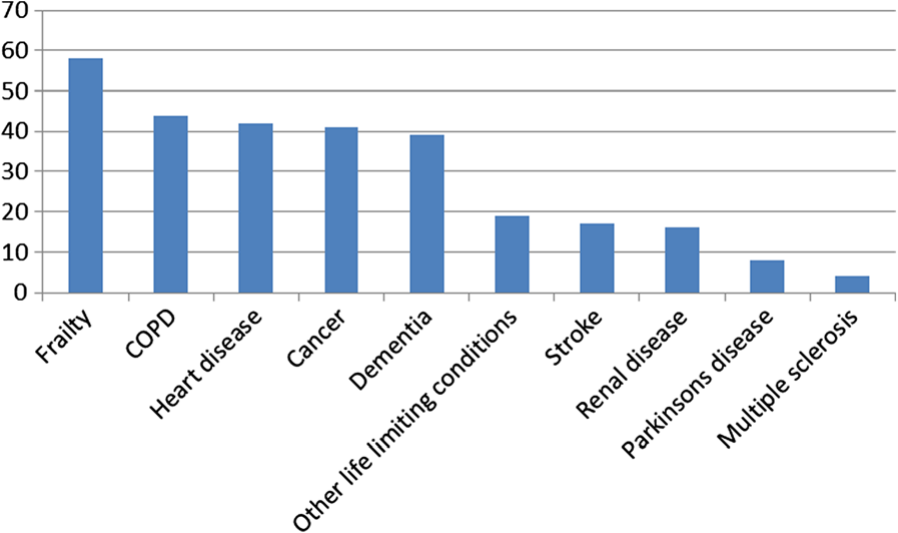
Total numbers of patients meeting each GSF prognostic indicator criteria (n = 208).

Figure 2 shows the breakdown of GSF criteria for the group of patients with palliative care needs (n = 208). The most prevalent GSF criteria was frailty (27.9%), followed by roughly equal numbers of patients with COPD (21.1%), heart disease (20.2%), cancer (19.7%), and dementia (18.8%).

Table 2 provides details of admission data for the group of patients with palliative care needs (n = 208).

**Table 2:** Admission data for patients with palliative care needs according to GSF criteria (n = 208)


Source of admission	A&E	128 (61.5%)
	GP	8 (3.8%)
	Clinic	4 (1.9%)
	Bed bureau	26 (12.5%)
	Other	18 (8.6%)
	[no data in notes]	24 (11.5%)
Time of admission to hospital	Normal working hours (9.00 – 17.00)	70 (33.6%)
	Out of hours (17.00 – 9.00)	138 (66.3%)
Reason for admission to hospital	Fall/confusion or deterioration	31 (14.9%)
	Cancer complication	29 (13.9%)
	COPD exacerbation	27 (13.0%)
	Chronic heart disease/HF exacerbation	13 (6.3%)
	Diabetes complication	8 (3.8%)
	Dementia complication	13 (6.3%)
	Stroke/TIA	11(5.2%)
	MI/heart surgery/acute cardiac event	14 (6.7%)
	Accidental injury	12 (5.8%)
	Renal failure	11 (5.2%)
	Infection	14 (6.7%)
	Neurological complication (Parkinsons, epilepsy, MS)	6 (2.9%)
	Other	19 (9.1%)

### Appropriateness of admissions

The majority of patients who met GSF criteria for palliative care need were admitted to hospital for appropriate reasons (n = 180, 86.5%). For only 14 (6.7%) patients was the admission considered to be potentially avoidable, 8 from RLI and six from SNGH. The measure of chance corrected agreement (quadratic weighted kappa on the ordered score) within the double coded sample indicate high levels of agreement between consultants (Kappa = 0.792, n = 30). Table 3 provides demographic information for the patients whose admission was considered potentially avoidable. All of these patients were older, with a median age of 84 years and an age range of 75 – 97 years. Half lived in nursing or residential care.

**Table 3:** Demographic information for patients whose admission was considered potentially avoidable (n = 14)


Sex	Male	7 (50%)
	Female	7 (50%)
Age	Median	84 years
	Age Range	75-97 years
Partnership status	Married	1 (7.1%)
	Divorced	1 (7.1%)
	Widowed	11 (71.4%)
	[no data in notes]	1 (7.1%)
Ethnic origin	White	14 (100%)
Living arrangements	Lives alone	3 (21.4%)
	Co-habits	3 (21.4%)
	Nursing home or residential care	7 (50%)
	[no data in notes]	1 (7.1%)

Table 4 provides admission and diagnostic data for the 14 patients whose admission was considered potentially avoidable. The route of admission for the majority of patients was via Accident& Emergency (A&E)/Emergency Department (n = 8), and most patients (n = 12) were admitted to hospital ‘out of hours’ (i.e. outside of 9.00 – 17.00, Monday – Friday).

**Table 4:** Diagnostic and admission data for patients whose admission was considered to be potentially avoidable (n = 14)


Source of admission	Clinic	1 (7.1%)
	A&E	8 (57.1%)
	GP	4 (28.6%)
	Other	1 (7.1%)
Time of admission	Usual working hours (9.00 – 17.00)	2 (14.3%)
	Out of hours (17.00 – 9.00)	12 (85.7%)
Reason for admission to hospital	Fall	2 (14.3%)
	Confusion/general deterioration	5 (35.7%)
	Urinary Tract Infection	1 (7.1%)
	Stroke	2 (14.3%)
	Intra-abdominal catastrophe	1 (7.1%)
	Pain/symptom control	3 (21.4%)
Underlying diagnosis	Cancer	6 (42.9%)
	Frailty	2 (14.3%)
	End stage renal failure	1(7.1%)
	Stroke	3 (2.1%)
	Dementia	1 (7.1%)
	Encephalopathy	1 (7.1%)
DNAR order in place		11 (78.6%)
Placed on Liverpool Care Pathway		1 (7.1%)
Evidence of referral to specialist palliative care		4 (28.6%)
Suggested alternative place of care	Hospice	3 (21.4%)
	Nursing home	10 (71.4%)
	Own home	1 (7.1%)

**Figure 3:**
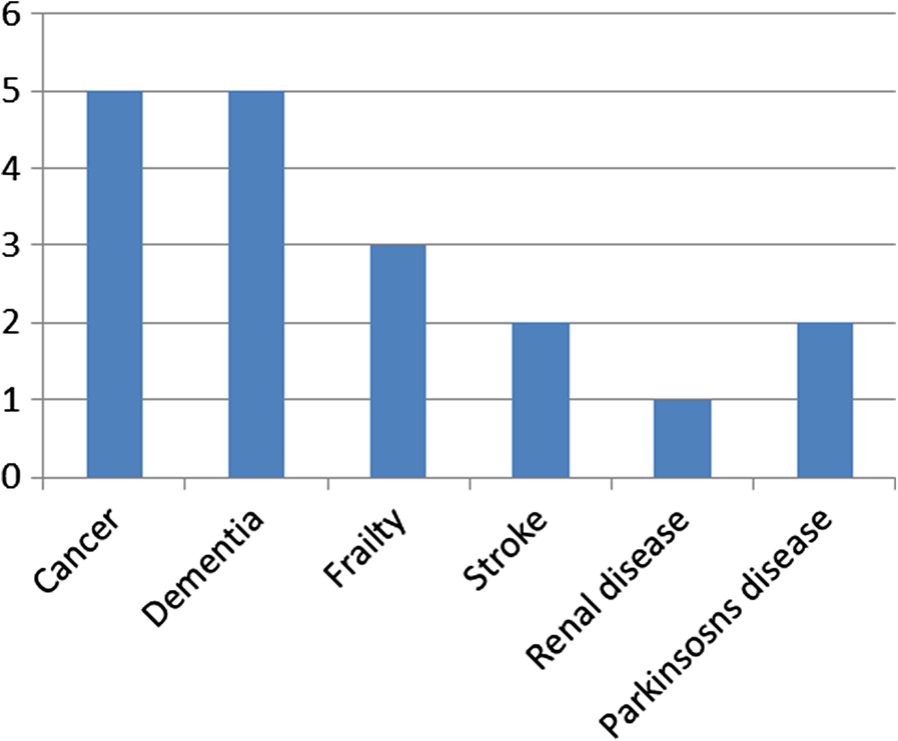
Numbers of patients meeting each GSF prognostic indicator, for patients whose admission was considered potentially avoidable (n = 14).

**Figure 4:**
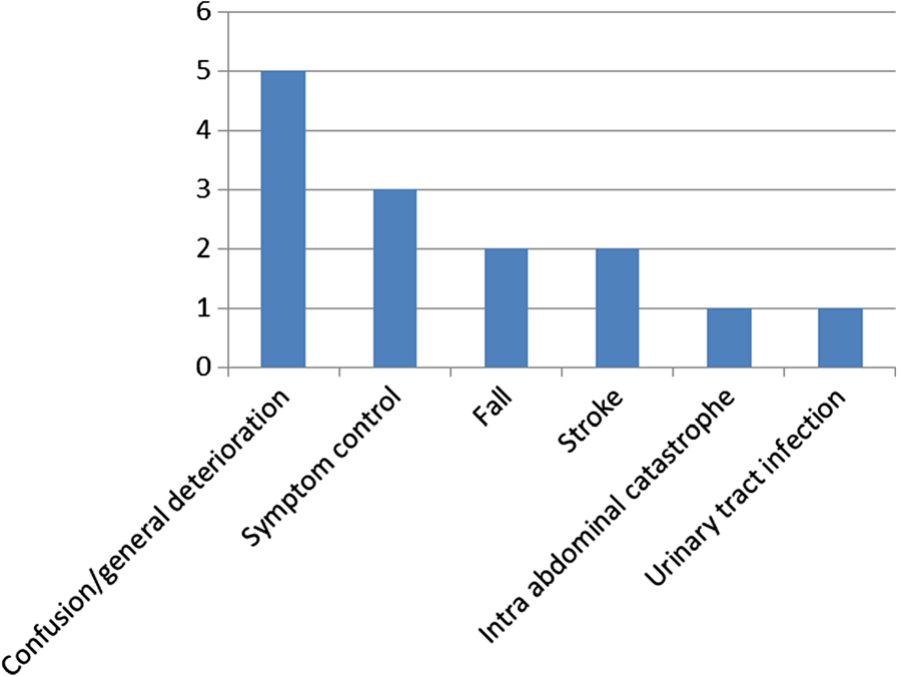
Reason for admission to hospital for potentially avoidable patients (n = 14).

Figure 3 provides a breakdown of the GSF prognostic indicator criteria that patients met, with cancer (n = 5), and dementia (n = 5) the most prevalent GSF criteria amongst the group of patients whose admission was considered potentially avoidable. Reason for admission to hospital is provided in Figure 4; most potentially avoidable admissions were for confusion/general deterioration (n = 5) or symptom control (n = 3). An appropriate alternative place of care was suggested for all admissions judged to be ‘potentially avoidable’ ; the most commonly suggested alternative was nursing or residential care (n = 10). Three patients could have been appropriately cared for in a hospice and one in their own home.

Of the 14 admitted patients whose admission was considered potentially avoidable, nursing staff questionnaires were available for 13 patients, and medical staff questionnaires were available for 10 patients. Tables 5 and 6 show summaries of responses given by nursing and medical staff regarding these patients. Nursing and medical staff recognised that most patients were in the last 12 months of life (70% and 90% respectively), however, when applying a standard definition [16], only around half of the patients were identified as having palliative care needs (50% and 54% respectively).

**Table 5:** Responses from questionnaires completed by nursing staff, regarding patients experiencing a potentially avoidable hospitalisation (n = 13)


	**Yes**	**No**
Would you be surprised if this patient died during the current admission?	5 (38.5%)	8 (61.5%)
Would you be surprised if this patient died within the next 12 months?	2 (15.4%)	10 (77.0%)
Do you believe this patient has palliative care needs?	7 (53.9%)	5 (38.5%)
Do you think this admission was clinically necessary?	7 (53.9%)	0

**Table 6:** Responses from questionnaires completed by medical staff, regarding patients experiencing a potentially avoidable hospitalisation (n = 10)


	**Yes**	**No**
Would you be surprised if this patient died during the current admission?	4 (40%)	6 (60%)
Would you be surprised if this patient died within the next 12 months?	1 (10%)	9 (90%)
Do you believe this patient has palliative care needs?	5 (50%)	5 (50%)
Do you think this admission was clinically necessary?	4 (40%)	1 (10%)

## Discussion

This study identified that, according to expert assessment, only 14 (6.7%) of 208 inpatient admissions amongst patients with palliative care needs in two acute settings in England were ‘potentially avoidable’. Those patients whose admissions were considered to be ‘potentially avoidable’ had a median age of 84. Half of the patients lived in residential or nursing care, and we concluded that most could have received care in this setting. The proportion of ‘potentially avoidable’ admissions identified is much lower than that suggested by previous research [10,11].Several reasons for this can be identified.

Firstly, and most importantly, admissions were considered by palliative medicine consultants from the standpoint of the admitting clinician and within the context of existing local health and social care services. In contrast, previous studies defined ‘appropriateness’ in relation to an ideal configuration of end of life care services, rather than the less than ideal situation that exists at the moment. A key strength of the study is the involvement of experienced local palliative medicine consultants in determining admission appropriateness who, crucially, have very good knowledge of the local configuration and extent of health and social care services for this patient group.

Secondly, their decision-making was also informed by the wider context of clinical risk and uncertainty that is critical to how these decisions are made in real-life acute situations. Two –thirds of the sample was admitted to hospital at a time when routine services might not be available. In contrast to previous studies [10,11], we also included a sample who were alive at the time of initial data collection and who met recognised diagnostic and prognostic criteria for palliative care need. We were also, therefore, able to include patients with palliative care needs who did not die during that hospital admission; this is important given the high number of hospital admissions many of these patients experience during the last year of life.

Whilst the absolute numbers of the group of patients classified as experiencing a ‘potentially avoidable’ hospitalisation was small, their socio-demographic and clinical characteristics do provide an interesting insight into where efforts to reduce potentially avoidable admissions would be most fruitfully expended. Most were older (median age 84 years) and lived in nursing or residential care. Crisis admission to hospital of nursing home residents remains an issue, with up to 67% of hospitalisations from nursing homes identified as avoidable [18]. There is evidence that older people in residential care settings receive variable quality end of life care, because of clinical and organisational factors [19]. A recent study which aimed to identify key factors influencing end of life care in nursing homes identified significant difficulties in implementing appropriate end of life care due to factors including lack of support from other agencies, lack of out of hours support, lack of access to training, variable support by general practitioners (GPs), and reluctance among GPs to prescribe appropriate medication [20]. In addition, high continuity of primary care has been shown to be important in reducing potentially avoidable hospitalizations amongst older people [21]. Whilst good Advance Care Planning (ACP) processes have the potential to help prevent admissions, where this is in line with the patient’s wishes, ACP remains under-developed within a UK context [22].

The majority of patients in this study accessed the hospital following presentation to the Accident & Emergency Department and were admitted out of hours. In the UK unplanned admissions have risen steadily over the past 10 years. Most of these ‘unplanned’ admissions occur ‘out of hours’, and most are admissions via A&E [23]. The King’s Fund Report (‘Avoiding hospital admissions’) found that many admissions labelled as ‘avoidable’ or ‘potentially avoidable’ are necessary as the only place that the services which are clinically required are available is in the acute sector [23]. The report found that certain interventions could reduce inpatient admissions; for example an emergency medicine consultant reviewing patients on admission could reduce inpatient admissions by 12% and specifically reduce admissions to the medical assessment unit by 21%. Whilst this evidence was generated from a general hospital population rather than from patients with palliative care needs, it is reasonable to assume that interventions of this nature would have a similar impact within a palliative care population. However, we are very far from being in the situation where a senior clinician is available to review every hospital admission and, again, many of these admissions could only be prevented if patients had good access to a comprehensive range of primary and community services.

### Limitations

There are a number of recognised limitations to this study. Assessments of potentially avoidable admissions were made by Palliative Medicine consultants from the standpoint of the admitting clinician, using expert judgements based on complete case note information. This subjective approach may be open to individual bias, however in the absence of any validated protocols to identify potentially avoidable admissions [17,18], context specific expert judgement was deemed the most appropriate way to classify admissions.

Seventy four patients were excluded as complete data were not available. Whilst there were no significant differences in age and gender between these patients and those who were included, other differences between the groups may exist. Therefore the generalisability of the dataset should be considered with caution.

Statistical comparisons between appropriate admissions and potentially avoidable admissions were not possible due to the small numbers of potentially avoidable admissions. Future research should seek to address this gap in current understanding in order to tailor interventions to those most at risk of potentially avoidable admissions.

## Conclusion

This study provides important insights into the issue of potentially avoidable hospital admissions amongst patients with palliative care needs in England. Our data challenge the assumption that, within the existing configuration and capacity of end of life care health and social care services, patients with palliative care needs experience a high level of potentially avoidable hospitalisations. Future research is needed adopting a similar ‘real world’ approach to defining potentially avoidable hospital use in a complete patient population. Also, more evidence is required about the resources needed to deliver the kind of community-based services that are likely to prevent potentially avoidable admissions to hospital amongst patients with palliative care needs, as well as a further understanding of the preferences of patients themselves and their families.

## Ethical approval

This study was approved by the Nottingham 1 Research Ethics Committee. Research Governance approval was granted by the relevant NHS Trusts.

## Competing interests

The authors declared that they have no competing interest.

## Authors’ contributions

MG and CI are co-principal investigators and designed the study. MG wrote the first draft. CI revised drafts of the paper. CG undertook data collection, analysis and revised drafts of the paper. JS is a co-investigator for the project and revised later drafts of the paper. BN and MB are co-investigators for the project and completed the appropriateness of admission assessment. MC is a co-investigator and commented on early drafts of the paper. All authors read and approved the final manuscript.

## Pre-publication history

The pre-publication history for this paper can be accessed here:

http://www.biomedcentral.com/1472-684X/12/9/prepub
